# Sex differences of brain cortical structure in major depressive disorder

**DOI:** 10.1093/psyrad/kkad014

**Published:** 2023-09-08

**Authors:** Jingping Mou, Ting Zheng, Zhiliang Long, Lan Mei, Yuting Wang, Yizhi Yuan, Xin Guo, Hongli Yang, Qiyong Gong, Lihua Qiu

**Affiliations:** Department of Radiology, the Second People's Hospital of Yibin, Yibin 644000, China; Department of Radiology, the Affiliated Hospital of Southwest Medical University, Luzhou 646000, China; Department of Radiology, the First People's Hospital of Yibin, Yibin 644000, China; Department of Radiology, the Affiliated Hospital of Southwest Medical University, Luzhou 646000, China; Sleep and NeuroImaging Center, Faculty of Psychology, Southwest University, Chongqing 400715, China; Department of Radiology, the Second People's Hospital of Yibin, Yibin 644000, China; Department of Radiology, the Second People's Hospital of Yibin, Yibin 644000, China; Department of Radiology, the Affiliated Hospital of Southwest Medical University, Luzhou 646000, China; Department of Radiology, the Second People's Hospital of Yibin, Yibin 644000, China; Department of Radiology, the Second People's Hospital of Yibin, Yibin 644000, China; Department of Radiology, the Affiliated Hospital of Southwest Medical University, Luzhou 646000, China; Department of Radiology, the Second People's Hospital of Yibin, Yibin 644000, China; Department of Radiology, the Affiliated Hospital of Southwest Medical University, Luzhou 646000, China; Huaxi MR Research Center (HMRRC), West China Hospital of Sichuan University, Chengdu 610041, China; Department of Radiology, the Second People's Hospital of Yibin, Yibin 644000, China; Department of Radiology, the Affiliated Hospital of Southwest Medical University, Luzhou 646000, China; Research Center of Neuroimaging big data, the Second People's Hospital of Yibin, Yibin 644000, China

**Keywords:** major depressive disorder, gender differences, magnetic resonance imaging, cortical thickness, surface area

## Abstract

**Background:**

Major depressive disorder (MDD) has different clinical presentations in males and females. However, the neuroanatomical mechanisms underlying these sex differences are not fully understood.

**Objective:**

The purpose of present study was to explore the sex differences in brain cortical thickness (CT) and surface area (SA) of MDD and the relationship between these differences and clinical manifestations in different gender.

**Methods:**

High-resolution T1-weighted images were acquired from 61 patients with MDD and 61 healthy controls (36 females and 25 males, both). The sex differences in CT and SA were obtained using the FreeSurfer software and compared between every two groups by *post hoc* test. Spearman correlation analysis was also performed to explore the relationships between these regions and clinical characteristics.

**Results:**

In male patients with MDD, the CT of the right precentral was thinner compared to female patients, although this did not survive Bonferroni correction. The SA of several regions, including right superior frontal, medial orbitofrontal gyrus, inferior frontal gyrus triangle, superior temporal, middle temporal, lateral occipital gyrus, and inferior parietal lobule in female patients with MDD was smaller than that in male patients (*P* < 0.01 after Bonferroni correction). In female patients, the SA of the right superior temporal (*r* = 0.438, *P* = 0.008), middle temporal (*r* = 0.340, *P* = 0.043), and lateral occipital gyrus (*r* = 0.372, *P* = 0.025) were positively correlated with illness duration.

**Conclusion:**

The current study provides evidence of sex differences in CT and SA in patients with MDD, which may improve our understanding of the sex-specific neuroanatomical changes in the development of MDD.

## Introduction

Major depressive disorder (MDD) is a significant threat to people's health, characterized by symptoms such as depression, cognitive impairment, loss of interest, and low energy (Song *et al*., 2018). It is predicted to become the leading cause of global disease burden by 2030 (Malhi and Mann, 2018). MDD is more common in women, with twice the prevalence compared to men from adolescence to adulthood (Nolen-Hoeksema, 2016). Hormonal changes, such as estrogen and progesterone imbalance in females and low testosterone levels in males, have been implicated in the sex differences in depression (Kundakovic and Rocks, 2022). Additionally, biological, psychological, and social-cultural factors contribute to the sex differences in depression (Hyde and Mezulis, 2020).

Clinical characteristics of MDD differ between males and females. Males with MDD often exhibit severe symptoms such as impulsivity, irritability, and insomnia, while females with MDD are more likely to present with somatic symptoms and atypical depression symptoms including increased appetite, weight gain, fatigue, and difficulty sleeping (Kim *et al*., 2015; Yang *et al*., 2017). Females also tend to have a better response to serotonergic antidepressants compared to males (Sramek *et al*., 2016).

Neuroimaging studies have shown differences in brain activity between female and male patients with MDD. Male patients with MDD exhibit lower amplitude of low-frequency fluctuation values in the left superior/middle frontal gyrus, and higher amplitude of low-frequency fluctuation values in the left postcentral gyrus when compared to female patients (Yao *et al*., 2014). Structural brain studies have also identified sex differences in gray matter volume (GMV) changes in individuals with MDD (Taki *et al*., 2005; Yang *et al*., 2017). GMV is determined by cortical thickness (CT) and surface area (SA), both of which have genetic and phenotypic independence, and reflect the different properties of gray matter structures (Grasby *et al*., 2020; Winkler *et al*., 2010). CT reflects the number of cells within a column, while SA reflects the number of cortical columns (Lee *et al*., 2021). Therefore, CT and SA measurements should be taken into consideration separately and prioritized over GMVs (Hanford *et al*., 2016; Winkler *et al*., 2010).

Most studies on sex-specific brain structure alterations in depression have focused on GMV (Kong *et al*., 2013; Yang *et al*., 2017), with limited research on CT and SA (Hu *et al*., 2022; Li *et al*., 2020). Thus, our study aimed to investigate the sex differences in CT and SA in individuals with MDD and explore the associations between these differences and clinical manifestations in different sexes.

## Methods

### Participants

This study involved patients diagnosed with MDD obtained from the Department of Psychiatry Clinic, West China Hospital of Sichuan University. All patients underwent a comprehensive interview conducted by two seasoned psychiatrists, following the diagnostic criteria for MDD as per the *Diagnostic and Statistical Manual of Mental Disorders, Fourth Edition* (DSM-IV). Additionally, their mental condition was assessed using the 17-item Hamilton Rating Scale for Depression (Hamilton Depression Rating Scale, HAMD-17). We included only those patients who were in depressive episodes and scored a minimum of 18 on the HAMD-17, had not received any antidepressant treatment for at least 1 year preceding the imaging examination, and did not have any other neurological or affective disorders.

We recruited the healthy control (HC) group through poster advertising. All volunteer participants underwent the SCID-I Non-Patient Edition scale to rule out the possibility of neuropsychiatric disorders. The exclusion criteria for all participants were as follows: (i) history of traumatic brain damage; (ii) organic brain lesions, such as brain tumors; (iii) alcohol or drug abuse (as per DSM-IV diagnostic criteria); (iv) pregnancy; and (v) neurological diseases such as epilepsy and multiple sclerosis.

Based on these inclusion and exclusion criteria, we recruited 61 patients with untreated MDD (25 male, 36 female) and 61 healthy volunteers (25 male, 36 female) in the study. An experienced radiologist confirmed the absence of abnormalities on conventional magnetic resonance imaging (MRI) scans for all participants. The Medical Ethics Committee of the Second People's Hospital of Yibin approved this study (no. 2014–056-01), and all participants provided written informed consent.

### MRI Data Acquisition

High-resolution anatomical images of the whole brain were obtained using a 3 Tesla MRI system (EXCITE, General Electric), equipped with an eight-channel phased-array head coil. We employed a 3D, sagittal, magnetization-prepared rapid gradient echo (MPRAGE) sequence to acquire three-dimensional T1-weighted images. The parameters used were: 156 axial slices; slice thickness, 1 mm; TR, 1900 ms; TE, 2.26 ms; flip angle, 12°; FOV, 240  × 240 mm; and data matrix, 256 × 256. All participants were fitted with foam padding and earplugs, and instructed to remain still during scanning (Yang *et al*., 2017).

### Data Preprocessing

The cortical surface of the 3D T1 image was constructed using the FreeSurfer software (http://surfer.nmr.mgh.harvard.edu/, v.5.3.0). This software measures the CT and SA of the entire cortex by automatically performing surface reconstruction, transformation, and high-resolution inter-individual calibration steps (Han *et al*., 2014; Muschelli *et al*., 2018). First, the original Digital Imaging and Communications in Medicine images for T1-weighted data are converted to the Neuroimaging Informatics Technology Initiative format, then to the MGZ format, and head movement correction is applied. Next, completing the affine transformation from the original volume to the Montreal Neurological Institute (MNI) 305 atlas and carrying out the Talairach coordinate system transformation. Then standardizing the signal strength of the original volume, performing the strength correction, and removing the deviation of the signal strength, and generating the original curved surface and performing automatic local anatomical correction, and expanding the generated cortical image and converting it to the spherical distribution template. The images were then smoothed using a 25-mm, full-width at half-maximum Gaussian kernel. The results from the automatic image processing for each subject were checked manually to assess whether the brain surface reconstruction was consistent with the grey matter boundary. If inconsistent, manual editing is performed. We obtained CT by calculating the shortest distance from the gray/white boundary to the gray/cerebrospinal fluid boundary at each vertex. The SA of each hemisphere is calculated by adding up the area of all tessellations on the gray matter surface (Deng *et al*., 2019; Qiu *et al*., 2014; Xiao *et al*., 2023).

### Statistical Analysis

We compared demographic and clinical data across the four groups (male MDD, female MDD, male HC, and female HC) using SPSS (v.25.0). Age and education years among groups were compared using a two-way analysis of covariance. The illness duration and HAMD score in male and female groups with MDD did not exhibit normal distributions and were analyzed as non-parametric using the Mann–Whitney *U*-test. CT and SA among the four groups were analyzed using a two-way analysis of covariance, taking sex (male, female) and diagnosis (MDD, HC) as between-participant factors, and intracranial volume as a covariate in MATLAB. The statistical results were adjusted using Bonferroni correction with a significance level of *P* < 0.01. We identified brain regions with sex differences in CT and SA among the four groups, which were then compared using a *post hoc* test (Student–Newman–Keuls method) between every two groups. The results were corrected using Bonferroni correction. The statistical threshold was set at *P* < 0.05. Spearman correlation analysis was performed to investigate the correlation of brain regions with sex differences in MDD and their clinical characteristics (illness duration, HAMD score), with the statistical significance threshold set at *P* < 0.05.

## Results

### Participants' Demographic and Clinical Characteristics

Participants' demographic and clinical characteristics are presented in Table 1. No significant differences were observed between the four groups in terms of age or years of education (*P* > 0.05). Similarly, there was no significant difference in HAMD score between male and female patients with MDD (*P* > 0.05). However, male patients with MDD had a longer illness duration than female patients (*P* < 0.05).

**Table 1:** Participants' demographic and clinical characteristics ($\bar {\rm x}$ ± s).

	Male MDD (*n* = 25)	Female MDD (*n* = 36)	Male HC (*n* = 25)	Female HC (*n* = 36)	*P* value
Age (years)	36.36 ± 12.79	36.08 ± 10.72	34.88 ± 11.89	36.61 ± 11.83	0.95
Education year (years)	8.8 ± 3.2	8.6 ± 2.6	9.1 ± 3.1	8.3 ± 2.1	0.746
HAMD score	22.32 ± 4.63	23.89 ± 4.45	–	–	0.099^b^
Illness duration (weeks)	101.24 ± 98.43	29.14 ± 42.51	–	–	<0.001^b^

a The *P* values were obtained by two-way analysis of variance tests.

b The *P* values were obtained by Mann–Whitney *U*-test.

### The Main Effect of Sex on CT and SA

**Figure 1:**
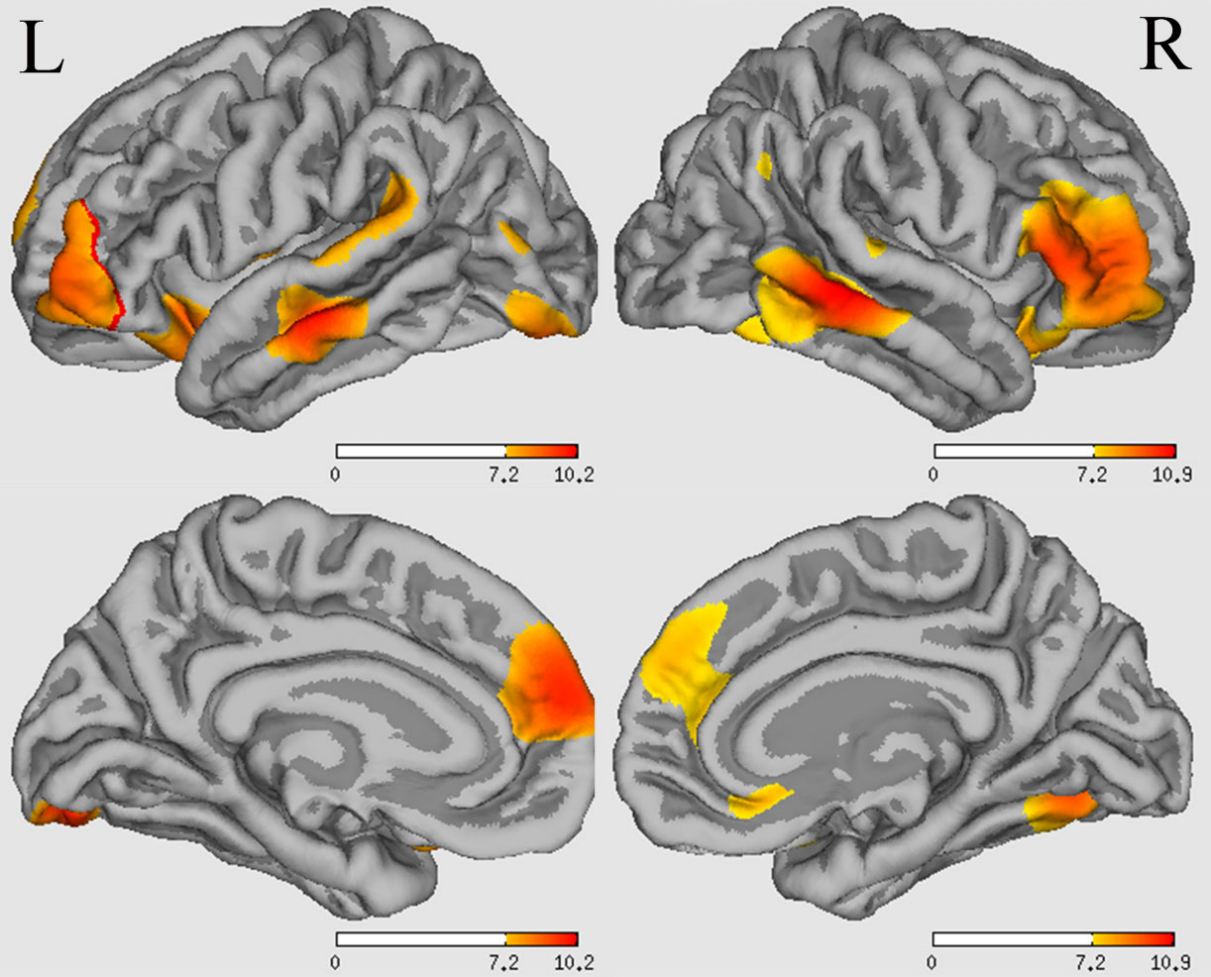
The main effect of sex difference on the SA of gray matter among four groups (L, left hemisphere; R, right hemisphere). The numbers on the color bars indicate −log *P* values. The warm color (red) indicates decreased SA in the female group (female MDD patients and female HCs) compared with the male group (male MDD patients and male HCs).

The effect of sex on CT was observed in the right precentral gyrus (*P* < 0.01 after FDR correction), but this was not maintained after applying the Bonferroni correction (a statistical threshold of *P* < 0.01). Refer to the Supplementary Material for further discussion and descriptions of the results. A significant effect of sex on SA was found in bilateral inferior frontal gyrus triangle, middle temporal, lateral occipital, left postcentral, rostral anterior cingulate, supramarginal gyrus, insula, right superior frontal, medial orbitofrontal, superior temporal gyrus, and inferior parietal lobule (Bonferroni correction, *P* < 0.01) (Fig. 1). On examining the effect of diagnosis and sex-diagnosis interactions on CT and SA, respectively, no cluster survives Bonferroni correction or FDR correction at a threshold of *P* < 0.01.

### *Post Hoc* Comparison Results

*Post hoc* analysis revealed that the CT of the right precentral gyrus in the male MDD group was thinner than that of the female MDD group (Supplementary Table S1). The SA of the right superior frontal, medial orbitofrontal gyrus, inferior frontal gyrus triangle, superior temporal, middle temporal, lateral occipital gyrus, and inferior parietal lobule in the female MDD group was smaller than that of the male MDD group (Table 2). The detailed results of pairwise comparisons of other groups are shown in the Supplementary Material.

**Table 2:** *Post hoc* test results showing the differences in SA in right hemisphere between the male and female MDD groups (mm^2^, $\bar {\rm x}$ ± s).

Region	M	F	size (mm^2^)	MNI (*x, y, z*)	*P* value
superior frontal gyrus	0.74 ± 0.13	0.66 ± 0.10	744	7, 50, 30	0.043
medial orbitofrontal gyrus	0.38 ± 0.05	0.35 ± 0.03	147	8, 27, −12	0.006
inferior frontal gyrus triangle	1.00 ± 0.10	0.93 ± 0.08	4268	53, 28, 5	0.021
superior temporal gyrus	0.63 ± 0.09	0.55 ± 0.06	230	59, −29, 8	0.001
middle temporal gyrus	0.70 ± 0.08	0.64 ± 0.06	1865	64, −35, −8	0.017
lateral occipital gyrus	1.00 ± 0.12	0.87 ± 0.10	889	29, −69, −8	<0.001
inferior parietal lobule	0.87 ± 0.18	0.77 ± 0.11	55	46, −59, 31	0.039

*x, y*, and *z* are the coordinates of the primary peak locations in the MNI space; M, male MDD group; F, female MDD group. Bonferroni correction; *P* < 0.05 is considered statistically significant.

### Correlation Analysis

**Figure 2:**
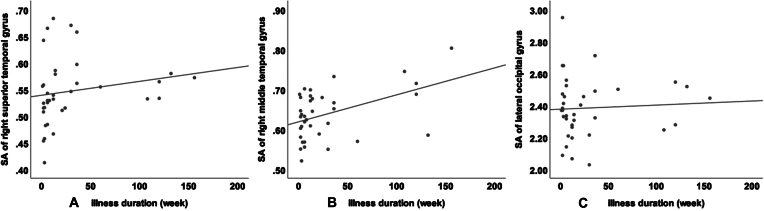
Correlation analysis in female MDD patients. (A) Correlation between the SA of the right superior temporal gyrus and illness duration. (B) Correlation between the SA of the right middle temporal gyrus and illness duration. (C) Correlation between the SA of the right lateral occipital gyrus and illness duration.

In female patients, a positive correlation was found between the SA of the right superior temporal (*r* = 0.438, *P* = 0.008, Fig. 2A), middle temporal (*r* = 0.340, *P* = 0.043, Fig. 2B), and lateral occipital gyrus (*r* = 0.372, *P* = 0.025, Fig. 2C) and illness duration. There were no significant correlations between the SA and HAMD score or illness duration in male patients with MDD.

## Discussion

Our study's findings indicate sex differences in the cortical structure of male and female patients with MDD. This may have implications for the clinical characteristics of depression in males and females. Specifically, we found thinner CT of the right precentral gyrus in males compared to females with MDD, although this difference did not survive Bonferroni's correction. Additionally, the SA of multiple brain regions was larger in males than in females with MDD, particularly in the frontotemporal and parietal cortices. The alterations of SA are more significant than CT, possibly due to the genetic and phenotypic independence of these measures. Our findings indicate that sex differences in CT and SA might be associated with the neurobiological processes underlying the different clinical characteristics of male and female patients with MDD.

### Sex Differences in Surface Area

We found significant differences in SA between male and female groups of MDD. These regions primarily involve the default mode network (DMN), frontoparietal network (right inferior frontal gyrus triangle, superior frontal, medial orbitofrontal gyrus, superior temporal, middle temporal gyrus, and inferior parietal lobule), and visual network (right lateral occipital gyrus), which are thought to be essential in the disease process of depression (Yao *et al*., 2014).

Decreased DMN connection is related to social dysfunction in people with severe depression (Vanderhasselt *et al*., 2013). The right ventrolateral prefrontal cortex (VLPFC), the superior temporal and middle temporal gyrus are important regions of the DMN, and play a role in rumination, self-referential processing, and emotional appraisal (Song *et al*., 2022; Yang *et al*., 2021; Zhou *et al*., 2020). It was found that GMV and SA decreased in the right VLPFC in patients with MDD, and the reduced GMV of the right VLPFC related to negative emotions such as sorrow, anxiety, and fatigue (Lener *et al*., 2016). Dysfunction of the temporal lobe cannot suppress the generation of negative emotions by the prefrontal limbic system, which ultimately manifests as persistent negative emotional experiences (Yu *et al*., 2017). In self-related negative events, patients with depression exhibit lower brain activity in the medial temporal lobe subsystem and increased brain activity in the dorsal medial prefrontal cortex subsystem of the DMN (Wang *et al*., 2022). In MDD, females pay greater attention to suicide, while males have a higher risk of successful suicide as they are more likely to succeed when committing killing themselves (Cavanagh *et al*., 2017). Cao *et al*. found that suicide attempters with MDD have alterations in intra- and inter-network connectivity between the DMN and right frontal-parietal network (Cao *et al*., 2020). Depressed patients with suicidality had abnormal cortical morphology in some brain regions within the DMN, frontolimbic circuitry, and temporal regions (Li *et al*., 2021a; Li *et al*., 2021b). When compared to suicide non-attempters, suicide attempters with MDD had smaller SA in the left superior frontal gyrus and larger SA in the left lateral occipital gyrus (Kang *et al*., 2020).

The right dorsolateral prefrontal cortex (DLPFC), and right inferior parietal lobule are acknowledged as important frontoparietal network-related areas and are linked to cognitive control, attention, and decision-making processes (Yao *et al*., 2014). Reduced functional connectivity (FC) in MDD has been observed between the DLPFC and the other areas of frontoparietal network (Ma *et al*., 2020; Yun and Kim, 2021). In MDD, antidepressant therapy was significantly associated with DLPFC abnormalities (Nestor *et al*., 2022; Zhukovsky *et al*., 2021). As networks implicated in the cognitive regulation of emotion, the frontoparietal network and subcortical regions, including the bilateral fusiform gyrus, are related to the state-dependent reconstruction of emotion regulation networks in individuals with MDD due to antidepressant treatment (Zhao *et al*., 2021). Males with MDD showed enhanced neural responses to acute psychosocial stress in the DLPFC and right frontoparietal network compared with females (Dong *et al*., 2022). The ENIGMA MDD Working Group's findings revealed that patients with more severe insomnia in MDD had smaller SA in part areas of the frontoparietal lobe and that only SA was predictive of the severity of insomnia in MDD, whereas GMV and CT had no predictive value (Leerssen *et al*., 2020). As evidenced by an increasing number of research, MDD-related suicide attempts are linked to the reduced SA and FC of the inferior parietal cortex (Campos *et al*., 2021; Chen *et al*., 2015; Li *et al*., 2022). The sex differences in SA we observed in the DMN and frontal-parietal network in individuals with MDD may associate with the different clinical symptoms (such as insomnia) and suicide risk between male and female patients with MDD.

The right lateral occipital gyrus is a region of the visual network, which is important in facial perception, expression, and emotional processing (Lee *et al*., 2021; Moreno-Ortega *et al*., 2019). Increased BOLD responses to sad stimuli in visual cortices may indicate effective antidepressant treatment (Furey *et al*., 2013; Keedwell *et al*., 2010; Moreno-Ortega *et al*., 2019). A recent article about the sex-specific SA and CT characteristics in MDD discovered that the alterations in the SA of the prefrontal cortex and the local gyrification index of the visual cortex were reversed in male and female MDD when compared to gender-matched HCs (Hu *et al*., 2022). In the visual networks, the occipital gyrus's diminished functional connectivity was connected to impaired visual processing in MDD (Lu *et al*., 2020). It has been discovered that the abnormal FC in the visual network was associated with the clinical symptoms of MDD (Wu *et al*., 2023). The alteration of visual cortical excitability was connected to the psychopathological characteristics of MDD (Du *et al*., 2022).

### Correlations with Illness Duration and HAMD Score

In female individuals with MDD, we observed a positive correlation between illness duration and the SA of the right superior temporal, middle temporal, and lateral occipital gyrus. The duration of depression is related to the degree of cognitive impairment (Pabel *et al*., 2018). MDD has been associated with cortical transcriptomic changes and there are sex differences in the effects of cortical gene expression on brain morphology (Miles *et al*., 2021). This study suggests that transcriptome-based polygenic score was associated with smaller amygdala volume and lower prefrontal gyrification across sexes in female patients, and related to hypergyrification in temporal and occipital areas in male patients. Another study reported that the decreased GMV in the right temporal gyrus was correlated with longer illness duration, and long-term sick patients had reduced GMV in the right superior and middle temporal gyrus compared to the early course cohort (Jiang *et al*., 2023). The temporal lobe is crucial for cognitive processing, including language, memory, and object vision processing (Davey *et al*., 2016). It has been reported that the thinner occipital cortex may be an endophenotype for MDD, and the response time to emotionally disturbing tasks in adolescents with depression was negatively correlated with the activation of the lateral occipital cortex (Colich *et al*., 2017). Hu *et al*. found that the higher local gyrification index in the left visual cortex was correlated with higher HAMD score in female patients with MDD, while this correlation was not observed in males with MDD (Hu *et al*., 2022). They also conducted a correlation analysis between the structural characteristics and the subscales of the HAMD scale, founding that higher local gyrification index in the left visual cortex was correlated with higher somatization score in female patients with MDD. Considering the limited sample size, we did not analyze the correlation between cortical structure with sex differences and the HAMD subscore group. These findings highlight the presence of sex-specific brain structural changes in MDD and provide neuroanatomical mechanisms for gender differences in clinical manifestations in MDD patients. Zacková *et al*. demonstrated that the reduced volume of the superior temporal gyrus may be related to communication deficits and infrequent participation in socially stimulating activities in MDD (Zacková *et al*., 2021).

### Limitations

Despite the strengths of our study, several limitations should be noted. First, the sample size was relatively small, which limits the further investigation of the relationship between sex-specific cortical changes and specific clinical manifestation. Second, since the incidence rate for women is higher than that of men, the number of included female patients was greater than male patients in our present study, and the imbalance of sample size between men and women may affect the statistical results. Third, the longer illness duration in the male MDD group compared to the female MDD group may have confounded our results, as illness duration has been shown to be associated with brain alterations in MDD. Finally, the study's cross-sectional design precluded us from examining changes in brain structure over time, highlighting the need for longitudinal studies with larger sample sizes.

## Conclusion

Overall, our study revealed sex differences in SA in patients with MDD. These findings are instrumental in exploring the sex-specific neuroanatomical mechanisms of clinical manifestations in patients with MDD.

## Supplementary Material

kkad014_Supplemental_File

## Data Availability

The original contributions presented in the study are included in the article/supplementary material, further inquiries can be directed to the corresponding author.
