# Supplementary material for: Sex differences of brain cortical structure in major depressive disorder
Source: Psychoradiology. 2023 Sep 8;3:kkad014. doi: 10.1093/psyrad/kkad014 (PMC10939343; doi:10.1093/psyrad/kkad014)
Supplement: kkad014_Supplemental_File [file kkad014_Supplemental_File.docx]

**Supplementary Material**

Sex-specific alterations of cortical thickness in major depressive disorder and the supplementary results of the post-hoc comparison of surface area

Jingping Mou^a,b,c,^ ^#^, Ting Zheng^b,#^, Lan Mei^a^, Zhiliang Long^d^, Yuting Wang^a,b^, Yizhi Yuan^a^, Xin Guo^a,b^, Hongli Yang^a,b^, Xinyu Hu^e^, Qiyong Gong^e^, Lihua Qiu^a,b,f^*

**Sex-specific alterations of cortical thickness in major depressive disorder**

Brain structure studies have found sex differences in gray matter volume (GMV) changes in individuals with MDD (Taki et al., 2005; Yang et al., 2017), and there are few studies on sex differences in cortical thickness (CT) in patients with MDD (Hu et al., 2022; Li et al., 2020). Here, we present our preliminary findings of sex-specific alterations of CT in 61 patients with MDD and 46 age, sex, and years of education-matched healthy controls (36 females and 25 males, both). We also explore the correlations between these brain regions and clinical characteristics.

FreeSurfer software (http://surfer.nmr.mgh.harvard.edu/, v. 5.3.0) was used to construct the cortical surface of the 3D T1 image. This software measures the cortical thickness (CT) of the entire cortex by automatically reconstructing the surface, transformation, and high-resolution inter-individual calibration steps. Briefly, the gray and white matter were segmented and used for cortical reconstruction. Then manually inspect segmented images and constructed surface. CT was measured by calculating the shortest distance from the grey/white boundary to the grey/cerebrospinal fluid (CSF) boundary at each vertex. The dates were warped and registered as average spherical space (fsaverage) and smoothened using 25-mm FWHM (Deng et al., 2019; Qiu et al., 2014).

**Statistical Analysis**

Two-way analysis of covariance was used to analyze the CT among the four groups, with sex (male, female) and diagnosis (MDD, HC) as the between-subject factors, and intracranial volume as covariates in MATLAB. The statistical results were adjusted using false discovery rates (FDR) correction and Bonferroni correction respectively with a significance level of p<0.01. The extracted brain regions with sex differences in CT among the four groups were compared using a post-hoc test (Student-Newman-Keuls method, S-N-K) between every two groups. The results were corrected using Bonferroni correction; p<0.05 was considered statistically significant. Spearman correlation analysis was used to analyze the correlation of brain regions with sex differences in MDD and their clinical characteristics (illness duration, HAMD score), the statistical significance was set at p<0.05.

**The main effect of sex difference on CT**

The main effect of sex difference on CT was observed in the right precentral gyrus after FDR correction (statistical threshold 0.01, Figure S1), but this cluster did not persist after Bonferroni correction (statistical threshold 0.01). When we analyze the main effect of diagnosis and sex-diagnosis interactions on CT respectively, no cluster survives Bonferroni’s correction or FDR correction at a threshold of p < 0.01.
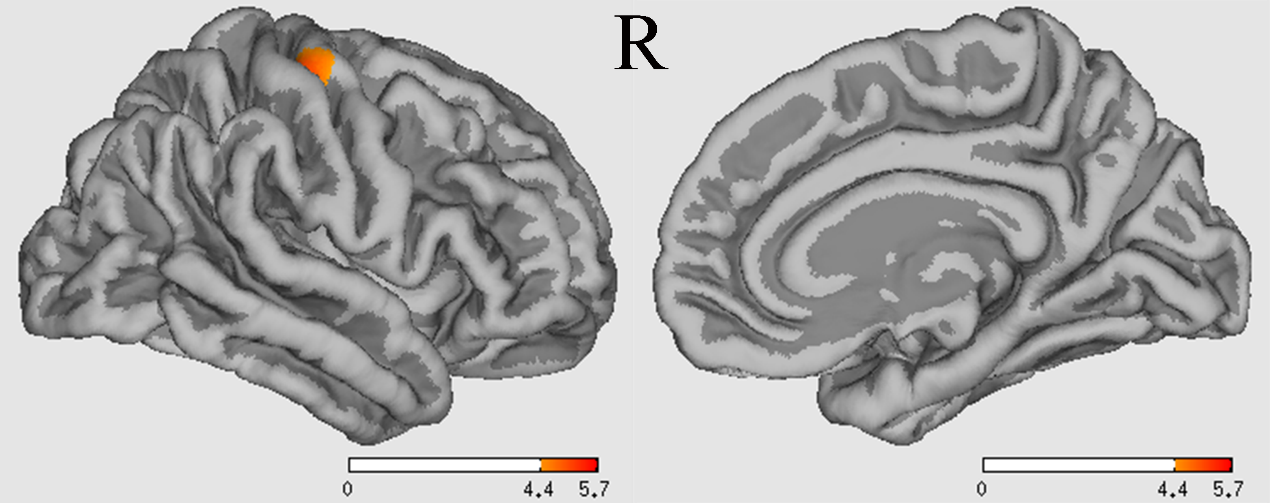


Figure S1. The main effect of sex difference on cortical thickness among four groups. R, right hemisphere. The numbers on the color bars indicate −log *p*-values. The warm color (red) indicates increased cortical thickness in the female group (female MDD patients and female healthy controls) compared with the male group (male MDD patients and male healthy controls). false discovery rates (FDR) correction, *p*＜0.01.

**Post-hoc comparison results of CT**

Post-hoc analysis revealed that the CT of the right precentral gyrus in the male MDD group was thinner than that of the female MDD group (Bonferroni correction; p<0.05, Table S1). There are significant differences in CT between the male HC group and female HC group (Table S2), and the male HC group and female MDD group (Table S3), the male MDD group and female HC group (Table S4).

Table S1. Post-hoc test results showing the differences in CT between the male and female MDD groups (mm, x̅±s)

| Region | Male MDD | Female MDD | size (mm2) | MNI (X, Y, Z） | *p*-value |
| --- | --- | --- | --- | --- | --- |
| right precentral gyrus | 2.22±0.12 | 2.35±0.15 | 282 | 33, −23, 53 | <0.001 |

MNI, Montreal Neurological Institute; X, Y, and Z are the coordinates of the primary peak locations in the MNI space; Bonferroni correction; p < 0.05 is considered statistically significant.

Table S2. Post-hoc test results showing the differences in CT between the male HC and female HC groups (mm, x̅±s)

| Region | Male HC | Female HC | size (mm2) | MNI (X, Y, Z） | *p*-value |
| --- | --- | --- | --- | --- | --- |
| right precentral gyrus | 1.02±0.12 | 0.90±0.08 | 280 | 33, −23, 53 | 0.001 |

MNI, Montreal Neurological Institute; X, Y, and Z are the coordinates of the primary peak locations in the MNI space; Bonferroni correction; p < 0.05 is considered statistically significant.

Table S3. Post-hoc test results showing the differences in CT between the male HC and female MDD groups (mm, x̅±s)

| Region | Male HC | Female MDD | size (mm2) | MNI (X, Y, Z） | *p*-value |
| --- | --- | --- | --- | --- | --- |
| right precentral gyrus | 0.61±0.11 | 0.55±0.06 | 280 | 33, −23, 53 | 0.003 |

MNI, Montreal Neurological Institute; X, Y, and Z are the coordinates of the primary peak locations in the MNI space; Bonferroni correction; p < 0.05 is considered statistically significant.

Table S4. Post-hoc test results showing the differences in CT between the male MDD and female HC groups (mm, x̅±s)

| Region | Male MDD | Female HC | size (mm2) | MNI (X, Y, Z） | *p*-value |
| --- | --- | --- | --- | --- | --- |
| right precentral gyrus | 2.22±0.12 | 2.33±0.14 | 280 | 33, −23, 53 | 0.022 |

MNI, Montreal Neurological Institute; X, Y, and Z are the coordinates of the primary peak locations in the MNI space; Bonferroni correction; *p* < 0.05 is considered statistically significant.

**Correlation analysis**

No significant correlations were observed between the CT of the right precentral gyrus and HAMD score or illness duration in either male or female individuals with MDD.

**Discussion**

We observed sex differences in the CT of the right precentral, although this result did not survive Bonferroni’s correction. Previous studies have reported reduced cortical thickness in the precentral gyrus (Papmeyer et al., 2015; Peng et al., 2015). The precentral gyrus is a crucial region of the frontal lobe that is responsible for voluntary movement and related to emotion regulation and cognitive processing (Seo et al., 2014). In patients with subthreshold depression, the volume of the precentral gyrus was reduced in elderly males, while it was not observed in females (Taki et al., 2005). Decreased activity in the precentral gyrus is linked to psychomotor symptoms and executive dysfunction (Guo et al., 2013). According to a near-infrared spectroscopy study, subjects with MDD exhibiting a history of suicidal conduct showed functional abnormalities in the precentral gyrus (Tsujii et al., 2017). In teenagers with MDD and suicidal thoughts, the ALFF of the right precentral gyrus was dramatically decreased after electroconvulsive therapy (Li et al., 2021). Female patients with MDD are more likely to attempt suicide, while male patients with MDD have a higher risk of successful suicide as they are more likely to succeed when committing suicide (Cavanagh et al., 2017). In this study, the CT of the right precentral gyrus of male patients with MDD was thinner than that of the female patients, which might be the anatomical basis for sex differences in suicide concept and behavior between male and female patients with MDD.

**Post-hoc comparison results of surface area (SA) between the male HC group and female HC group (Table S5), the male HC group and female MDD group (Table S6), and the male MDD group and female HC group (Table S7).**

Table S6. Post-hoc test results showing the differences in SA between the male HC and female HC groups (mm², x̅±s)

| Region（right hemisphere） | Male MDD | Female HC | size (mm2) | MNI (X, Y, Z） | *p*-value |
| --- | --- | --- | --- | --- | --- |
| inferior parietal gyrus | 0.87±0.18 | 0.75±0.11 | 282 | 33, −23, 53 | 0.006 |
| lateral occipital gyrus | 1.00±0.12 | 0.86±0.11 | 889 | 29, -69, -8 | <0.001 |
| medial orbitofrontal gyrus | 0.38±0.05 | 0.35±0.03 | 147 | 8, 27, -12 | 0.03 |
| middle temporal gyrus | 0.38±0.08 | 0.64±0.07 | 1865 | 64, -35, -8 | 0.014 |
| inferior frontal gyrus triangle | 1.00±0.10 | 0.90±0.08 | 4268 | 53, 28, 5 | <0.001 |
| superior frontal gyrus | 0.74±0.13 | 0.63±0.10 | 744 | 7, 50, 30 | 0.001 |
| superior temporal gyrus | 0.63±0.09 | 0.56±0.06 | 230 | 59, -29, 8 | 0.020 |

MNI, Montreal Neurological Institute; X, Y, and Z are the coordinates of the primary peak locations in the MNI space; Bonferroni correction; p < 0.05 is considered statistically significant.

Table S7. Post-hoc test results showing the differences in SA between the male HC and female MDD groups (mm², x̅±s)

| Region (right hemisphere) | Male HC | Female MDD | size (mm2) | MNI (X, Y, Z） | *p*-value |
| --- | --- | --- | --- | --- | --- |
| medial orbitofrontal v | 0.38±0.04 | 0.36±0.03 | 147 | 8, 27, -12 | 0.01 |
| middle temporal gyrus | 0.70±0.09 | 0.64±0.06 | 1865 | 64, -35, -8 | 0.029 |
| inferior frontal gyrus triangle | 1.02±0.12 | 0.93±0.08 | 4268 | 53, 28, 5 | 0.002 |
| superior temporal gyrus | 0.61±0.11 | 0.55±0.06 | 230 | 59, -29, 8 | 0.007 |

MNI, Montreal Neurological Institute; X, Y, and Z are the coordinates of the primary peak locations in the MNI space; Bonferroni correction; p < 0.05 is considered statistically significant.

Table S8. Post-hoc test results showing the differences in SA between the male MDD and female HC groups (mm², x̅±s)

| Region (right hemisphere) | Male MDD | Female HC | size (mm2) | MNI (X, Y, Z） | *p*-value |
| --- | --- | --- | --- | --- | --- |
| inferior parietal gyrus | 0.87±0.18 | 0.75±0.11 | 282 | 33, −23, 53 | 0.006 |
| lateral occipital gyrus | 1.00±0.12 | 0.86±0.11 | 889 | 29, -69, -8 | <0.001 |
| medial orbitofrontal gyrus | 0.38±0.05 | 0.35±0.03 | 147 | 8, 27, -12 | 0.03 |
| middle temporal gyrus | 0.38±0.08 | 0.64±0.07 | 1865 | 64, -35, -8 | 0.014 |
| inferior frontal gyrus triangle | 1.00±0.10 | 0.90±0.08 | 4268 | 53, 28, 5 | <0.001 |
| superior frontal gyrus | 0.74±0.13 | 0.63±0.10 | 744 | 7, 50, 30 | 0.001 |
| superior temporal gyrus | 0.63±0.09 | 0.56±0.06 | 230 | 59, -29, 8 | 0.020 |

MNI, Montreal Neurological Institute; X, Y, and Z are the coordinates of the primary peak locations in the MNI space; Bonferroni correction; p < 0.05 is considered statistically significant.

**References**

Cavanagh A, Wilson C J, Kavanagh D J, et al. (2017). Differences in the expression of symptoms in men versus women with depression: a systematic review and meta-analysis. Harv Rev Psychiatry [J], 25: 29-38.

Deng D M, Chen L Z, Li Y W, et al. (2019). Cortical morphologic changes in recent-onset, drug-naïve idiopathic generalized epilepsy. Magn Reson Imaging [J], 61: 137-142.

Guo W, Liu F, Xue Z, et al. (2013). Decreased interhemispheric coordination in treatment-resistant depression: a resting-state fMRI study. Plos One [J], 8: e71368.

Li X, Yu R, Huang Q, et al. (2021). Alteration of whole brain ALFF/fALFF and degree centrality in adolescents with depression and suicidal ideation after electroconvulsive therapy: a resting-state fMRI study. Front Hum Neurosci [J], 15: 762343.

Papmeyer M, Giles S, Sussmann J E, et al. (2015). Cortical Thickness in Individuals at High Familial Risk of Mood Disorders as They Develop Major Depressive Disorder. Biol Psychiatry [J], 78: 58-66.

Peng D, Shi F, Li G, et al. (2015). Surface vulnerability of cerebral cortex to major depressive disorder. Plos One [J], 10: e0120704.

Qiu L, Huang X, Zhang J, et al. (2014). Characterization of major depressive disorder using a multiparametric classification approach based on high resolution structural images. J Psychiatry Neurosci [J], 39: 78-86.

Seo D, Olman C A, Haut K M, et al. (2014). Neural correlates of preparatory and regulatory control over positive and negative emotion. Soc Cogn Affect Neurosci [J], 9: 494-504.

Taki Y, Kinomura S, Awata S, et al. (2005). Male elderly subthreshold depression patients have smaller volume of medial part of prefrontal cortex and precentral gyrus compared with age-matched normal subjects: a voxel-based morphometry. J Affect Disord [J], 88: 313-320.

Tsujii N, Mikawa W, Tsujimoto E, et al. (2017). Reduced left precentral regional responses in patients with major depressive disorder and history of suicide attempts. Plos One [J], 12: e0175249.

Yang X, Peng Z, Ma X, et al. (2017). Sex differences in the clinical characteristics and brain gray matter volume alterations in unmedicated patients with major depressive disorder. Sci Rep [J], 7: 2515.
